# Targeted Plasma Bile Acid Metabolomic Analysis in Metabolic Dysfunction-Associated Steatohepatitis and Alcoholic Hepatitis

**DOI:** 10.3390/biomedicines13010078

**Published:** 2024-12-31

**Authors:** Yuta Hirata, Yasunaru Sakuma, Hideo Ogiso, Ryozo Nagai, Kenichi Aizawa

**Affiliations:** 1 Division of Gastroenterological, Department of Surgery, General and Transplant Surgery, Jichi Medical University, Shimotsuke 329-0498, Tochigi, Japan; 2 Division of Clinical Pharmacology, Department of Pharmacology, Jichi Medical University, Shimotsuke 329-0498, Tochigi, Japan; 3 Jichi Medical University, Shimotsuke 329-0498, Tochigi, Japan; 4 Clinical Pharmacology Center, Jichi Medical University Hospital, Shimotsuke 329-0498, Tochigi, Japan; 5 Division of Translational Research, Clinical Research Center, Jichi Medical University Hospital, Shimotsuke 329-0498, Tochigi, Japan

**Keywords:** metabolic dysfunction-associated steatohepatitis, alcoholic hepatitis, bile acids, liver cirrhosis, biomarkers, mass spectrometry, fibrosis index, Child–Pugh score, MELD score

## Abstract

**Background:** Even though many metabolic liver diseases can now be diagnosed using blood tests and diagnostic imaging, early diagnosis remains difficult. Understanding mechanisms contributing to the progression from Metabolic Dysfunction-Associated Steatohepatitis (MASH) and Alcoholic Hepatitis (AH) to cirrhosis is critical to reduce the burden of end-stage liver disease. Monitoring individual bile acids has been proposed as a way to distinguish various liver disorders. **Methods:** This study explored bile acid profiles in patients with MASH and AH. Plasma samples from patients with MASH, AH, and a control group were analyzed using liquid chromatography-tandem mass spectrometry (LC-MS/MS) to quantify bile acid concentrations. Targeted metabolomic analysis was performed to compare bile acid levels between the hepatitis and control groups. **Results:** Concentrations of ursodeoxycholic acid (UDCA), chenodeoxycholic acid (CDCA), taurocholic acid (TCA), tauroursodeoxycholic acid (TUDCA), taurochenodeoxycholic acid (TCDCA), glycoursodeoxycholic acid (GUDCA), glycochenodeoxycholic acid (GCDCA), and glycocholic acid (GCA) were significantly elevated in the hepatitis group. Correlation analysis revealed strong positive relationships between the total and direct bilirubin levels and TUDCA and GCDCA. Aspartate aminotransferase (AST) showed strong positive correlations with TCDCA and GCDCA. Child–Pugh score, Fibrosis-4 index, and non-alcoholic fatty liver disease fibrosis score were positively correlated with GCA, whereas the aspartate aminotransferase-to-platelet ratio correlated with TCA, TCDCA, and GCA. The model for end-stage liver disease (MELD) score showed a strong positive correlation with GCDCA. **Implications:** GCDCA may serve as a predictive biomarker for liver damage, potentially enabling early diagnosis and targeted intervention in patients with MASH and AH.

## 1. Introduction

As Metabolic Dysfunction-Associated Steatotic Liver Disease (MASLD) and Alcohol-associated liver disease (ALD) increase worldwide [1,2], both cirrhotic diseases increasingly require liver transplantation [3,4]. MASLD, a liver disease in obesity and metabolic syndrome, is caused by excessive triglyceride accumulation in liver parenchymal cells [5]. It has been reported that 20% of patients with metabolic dysfunction-associated steatohepatitis (MASH) rapidly develop fibrosis and progress to liver failure within a few years [6]. The number of patients with ALD is also gradually increasing in the U.S., especially among patients younger than 45 years, who account for 30.2% of MASLD patients and 58.5% of deaths due to alcohol-related cirrhosis [2]. Although many metabolic liver diseases can now be diagnosed using blood tests and diagnostic imaging, early diagnosis remains difficult. In MASLD and ALD, parts of the enterohepatic circulatory system comprising the bile ducts, intestinal tract, portal vein, and liver are thought to be compromised, resulting in altered bile acid dynamics and an aberrant bile acid pool. In recent years, monitoring individual bile acids has been proposed to distinguish various liver disorders. A method for simultaneous analysis of primary and secondary bile acids has been developed for diagnosis of various diseases and is now being employed clinically [7,8,9,10,11].

ALD pathogenesis is mediated by three alcohol metabolic pathways: alcohol dehydrogenase (ADH), the microsomal ethanol oxidation system (MEOS) centered on cytochrome P4502El (CYP2E1), and the catalase pathway. In addition to cellular damage caused by acetaldehyde and reactive oxygen species, the intestinal microbiota is also important [12,13]. Metabolomic studies of ALD feces have shown that gut microbial composition and metabolism determine the prognosis of ALD, further demonstrating the importance of the enterohepatic linkage [14]. Enterobacteria alter the composition of bile acids via deconjugation and dehydrogenation. This interaction affects host metabolism. Studies in sterile and antibiotic-treated mice and rats have shown that the gut microbiota has a profound effect on bile acid diversity and pool size [15,16]. Drinking habits also alter the composition of the bacterial flora, and *Enterococcus faecalis* is increased in the stools of patients with ALD, especially alcoholic hepatitis (AH), and cytolysin produced by some strains of *E. faecalis* is associated with the severity of AH [17].

MASH is diagnosed based on fatty liver found in liver histology or imaging studies and excludes liver diseases other than MASLD. It is defined as the presence of 5% hepatic steatosis and inflammation with hepatocyte injury, e.g., ballooning, with or without fibrosis. This can progress to cirrhosis, liver failure, and, albeit rarely, liver cancer [18]. In MASH and AH, metabolomic analysis to identify abnormalities in bile acids should reveal a new strategy to diagnose early stages of metabolic disfunction.

Bile acids are broadly classified into primary bile acids, synthesized directly from cholesterol in the liver, and secondary bile acids, dehydroxylated by intestinal bacteria after primary bile acids are excreted into the small intestine. The primary bile acids are cholic acid (CA) and chenodeoxycholic acid (CDCA), and the secondary bile acids deoxycholic acid (DCA) and lithocholic acid (LCA), which are dehydroxylated from each other. In the bile of healthy individuals, CA, CDCA, and DCA comprise the majority of the bile [19]. The hydrophilicity and hydrophobicity of various bile acids vary depending on their structures, and LCA, DCA, and CDCA are considered highly hydrophobic and cytotoxic among bile acids [20].

Identifying mechanisms and causes of the transition from MASH and AH to cirrhosis is crucial in reducing the number of patients with cirrhosis. This study sought to identify new plasma biomarkers to improve early detection of liver damage in patients with MASH and AH. While these markers may not directly reveal the underlying mechanisms, they offer valuable insights into disease progression and have significant potential to enhance early diagnosis and targeted intervention, ultimately contributing to better patient outcomes.

## 2. Materials and Methods

### 2.1. Patients

The 11 patients in the hepatitis group included 3 with AH and 8 with MASH who underwent living donor liver transplantation from August 2022 to April 2024. The control group included 8 patients who underwent abdominal surgery from December 2023 to June 2024.

### 2.2. Blood Sample Collection

The blood samples were collected at the time of surgery for both groups, and the plasma samples were stored at −80 °C.

### 2.3. Chemicals and Reagents

Methanol, acetonitrile, ultra-pure water, and formic acid were of liquid chromatography-tandem mass spectrometry (LC-MS/MS) grade. These reagents were purchased from FUJIFILM Wako Pure Chemical (Osaka, Japan). Bile acids were purchased from the following manufacturers. Ursodeoxycholic acid (UDCA) was from FUJIFILM Wako Pure Chemical (Cat No: 357-25861). LCA, CA, DCA, CDCA, tauroursodeoxycholic acid (TUDCA), glycochenodeoxycholic acid (GCDCA), taurodeoxycholic acid (TDCA), glycocholic acid (GCA), and taurocholic acid (TCA) were from Nacalai Tesque (Cat No: 09174-54, 08843-14, 10711-64, 07938-01, 32731-24, 17132-44, 32740-91, 17123-51 and 32729-61, respectively; Kyoto, Japan). Glycodeoxycholic acid (GDCA) was from Toronto Research Chemicals (Cat No: G641400; North York, ON, Canada). Glycoursodeoxycholic acid (GUDCA) and 7-Ketolithocholic acid were from MedChemExpress (Cat No: HY-N1424 and HY-W018512, respectively; South Brunswick, NJ, USA). Taurochenodeoxy cholic acid (TCDCA) was from Cayman Chemical (Cat No: 20275; Ann Arbor, MI, USA).

### 2.4. Standard Preparation

Bile acid standard solutions were prepared at 1 mg/mL in methanol and stored at −80 °C. A solution of all standards was prepared by diluting them with methanol. A 17-point calibration curve was prepared by serial dilution of the mixture. An absolute calibration curve method was used to quantitatively analyze each bile acid in plasma. The lowest and highest points on the calibration curve were 1 and 1000 pmol/mL. The limit of quantification for each bile acid was less than 2 pmol/mL. Given that the plasma was diluted 20-fold in this study, the limit of quantitation for each bile acid in plasma was less than 40 pmol/mL.

### 2.5. Bile Acid Extraction

Ten μL of plasma or the standard solution was mixed with 40 μL of methanol. Extracts were then centrifuged (14,000× *g* for 5 min at 4 °C), after which 30 μL of the upper layers was collected in 1 mL polypropylene vials. Extracts were diluted 20-fold with 570 μL of methanol.

### 2.6. Targeted Bile Acid Analysis Using LC-MS/MS

Liquid chromatography tandem quadrupole mass spectrometry (LC-MS/MS) analysis was performed on an LCMS8060 system (Shimadzu, Kyoto, Japan). The bile acids were separated on a YMC-Triart C_18_ column (50 mm × 2.0 mm, 1.9 μm; YMC, Kyoto, Japan). The mobile phases A and B consisted of 0.1% (*v*/*v*) formic acid in water and 0.1% (*v*/*v*) formic acid in methanol/acetonitrile (1:1, *v*/*v*), respectively. The initial condition was 25% B. The following solvent gradient was employed: 25% B was followed by a linear gradient to 50% B from 0 to 2 min, to 85% B from 2 to 11 min, to 100% B from 11 to 11.5 min, and then held for 13.5 min. Subsequently, the mobile phase was returned to 25% B over 0.1 min and was maintained for 2.4 min until the end of the run. The oven temperature was 50 °C, and the flow rate was 0.35 mL/min. The sample volume injected was 4 µL.

The targeted analytes were detected in multi-reaction monitoring (MRM) mode using electrospray ionization. The MS parameters were used with default settings as follows: interface voltage of 4.0 kV (pos) and −3.0 kV (neg), nebulizer gas flow of 1.5 L/min, heating gas flow of 5 L/min, drying gas flow of 10 L/min, heat block temperature at 400 °C, and DL temperature at 250 °C. The mass transitions and parameters are shown in Table S1. The data were collected using LabSolutions software version 5.123 for LCMS8060 (Shimadzu). The concentration of each bile acid was determined from its peak area.

### 2.7. Liver Reserve Capacity and Liver Fibrosis Assessment Score

We examined the association between the two groups using the Child–Pugh (CP) score, the fibrosis-4 (FIB-4) index, the NAFLD (=MASLD) fibrosis score (NFS), the aspartate aminotransferase to platelet ratio index (APRI), albumin-bilirubin (ALBI) grade, and the model for end-stage liver disease (MELD) score. The CP score is one of the most widely used liver reserve assessment methods. It is calculated using five factors and evaluated on a three-point scale [21,22]. The FIB-4 index is one of the formulas to predict liver fibrosis and is calculated based on age, AST, ALT, and PLT [23,24]. The NFS is a liver fibrosis risk score for MASLD [25]. APRI combines AST, ALT, and platelet counts to assess the degree of fibrosis [26,27]. ALBI grade is an assessment of liver reserve for hepatocellular carcinoma. It is characterized by its ability to calculate liver reserve with only three common blood samples, albumin and total bilirubin, using a statistical approach [28,29]. The MELD score has been used at UNOS since 2002 to assess liver reserve capacity in liver transplant registrants aged12 years and older [30,31].

### 2.8. Statistical Analysis

The significance of differences between the two groups was evaluated using the Mann–Whitney U test. We used the U test and Pearson’s correlation coefficient for statistical analyses. The statistical results were expressed as median values. All statistical analyses were performed using SPSS, and *p* < 0.05 was considered significant.

## 3. Results

### 3.1. Hepatitis and Control Between-Group Comparisons

#### 3.1.1. Clinical Data Characteristics

The clinical characteristics of the MASH, AH, and control groups were compared. Diabetes mellitus, ursodeoxycholic acid administration, TBIL, DBIL, and AST were significantly higher in the hepatitis group. PT-INR was significantly prolonged in the hepatitis group. With respect to liver reserve capacity and the degree of liver damage, the CP score, FIB-4 index, NFS, APRI, ALBI grade, and MELD score were all significantly higher in the hepatitis group (Table 1). On the other hand, TCHOL was significantly lower in the hepatitis group (Table 1).

#### 3.1.2. Plasma Bile Acids Analyses

The following plasma bile acids were higher in the hepatitis group: UDCA, CDCA, TCA, TUDCA, TCDCA, GUDCA, GCDCA, and GCA (Table 2).

#### 3.1.3. Bile Acid and Blood Test Correlations

**Figure 1:**
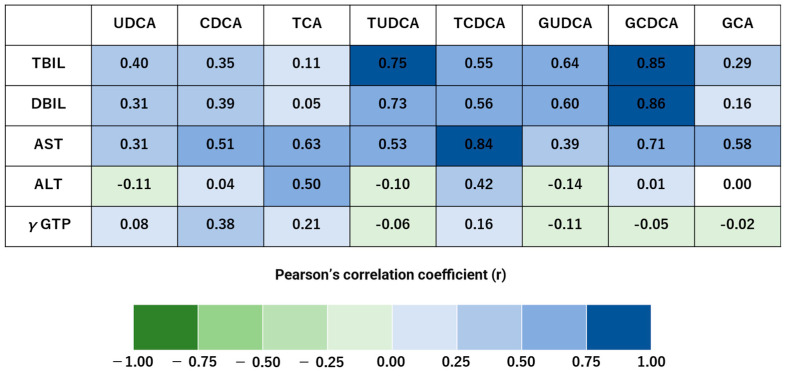
Specific bile acids show strong positive correlations with liver damage markers in the hepatitis and control groups. Pearson’s correlation coefficients of the 19 blood samples are visualized as heat maps, highlighting significant differences between the hepatitis and control groups. The correlation strength is classified as follows: 0.7 < |r| < 1.0 indicates a strong correlation. Total bilirubin (TBIL), direct bilirubin (DBIL), and aspartate transaminase (AST) were strongly and positively correlated with tauroursodeoxycholic acid (TUDCA) and glycochenodeoxycholic acid (GCDCA).

**Figure 2:**
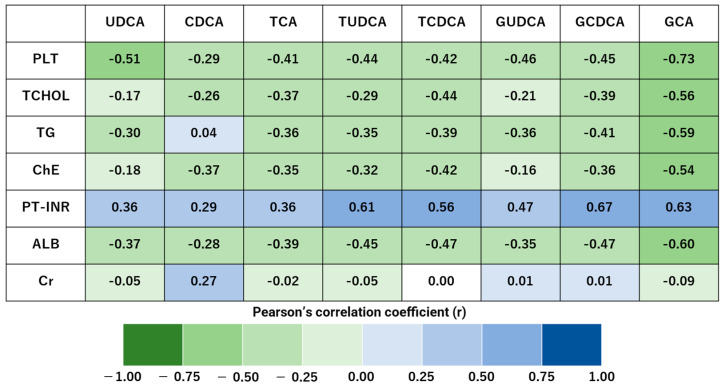
The glycocholic acid levels are strongly and negatively correlated with platelet counts in the hepatitis and control groups. Pearson’s correlation coefficients of the 19 blood samples are visualized as heat maps, highlighting significant differences between hepatitis and control groups. The correlation strength is classified as follows: 0.7 < |r| < 1.0 indicates a strong correlation. Platelet count (PLT) was strongly negatively correlated with glycocholic acid (GCA).

In both the hepatitis and control groups, there were strong correlations between TBIL, DBIL, and AST with TUDCA and GCDCA (Figure 1). PLT was strongly negatively correlated with GCA (Figure 2).

#### 3.1.4. Correlations Between Bile Acids, Liver Reserve, and Liver Fibrosis

**Figure 3:**
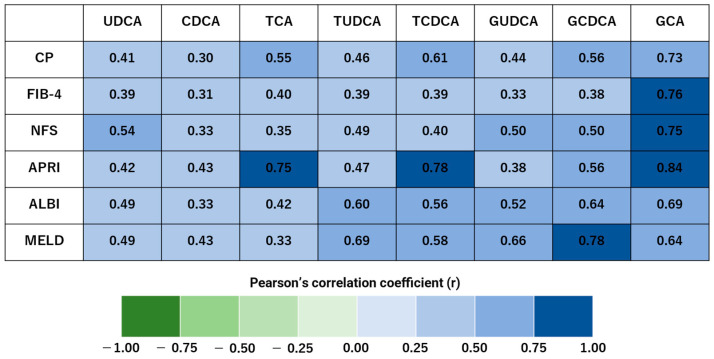
Bile acids show strong positive correlations with liver damage indicators in the hepatitis and control groups. Pearson’s correlation coefficients of the 19 blood samples are visualized as heat maps, illustrating significant relationships between bile acids, liver reserve, and liver fibrosis. The correlation strength is classified as follows: 0.7 < |r| < 1.0 indicates a strong correlation. Child–Pugh (CP), fibrosis-4 (FIB-4), and non-alcoholic fatty liver disease (NAFLD, also known as MASLD) fibrosis score (NFS) showed strong positive correlations with glycocholic acid (GCA). Similarly, the aspartate aminotransferase-to-platelet ratio index (APRI) was strongly correlated with taurocholic acid (TCA), taurochenodeoxycholic acid (TCDCA), and GCA. The model for end-stage liver disease (MELD) showed a strong positive correlation with glycochenodeoxycholic acid (GCDCA).

In the hepatitis and control groups, CP, FIB-4, and NFS showed a strong positive correlation with GCA, as did APRI with TCA, TCDCA, and GCA. MELD displayed a strong positive correlation with GCDCA (Figure 3).

#### 3.1.5. Receiver Operating Characteristic (ROC) Curves for Bile Acids Between the Hepatitis and Control Groups

**Figure 4:**
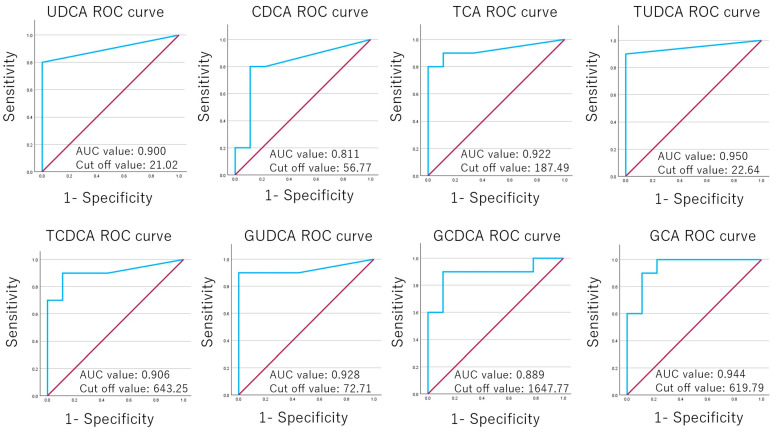
A total of 8 bile acids with under the curve (AUC) value >0.700 are shown. The AUC values for ursodeoxycholic acid (UDCA), chenodeoxycholic acid (CDCA), taurocholic acid (TCA), tauroursodeoxycholic acid (TUDCA), taurochenodeoxycholic acid (TCDCA), glycoursodeoxycholic acid (GUDCA), glycochenodeoxycholic acid (GCDCA), and glycocholic acid (GCA) were 0.900, 0.811, 0,922, 0.950, 0.906, 0.928, 0.889 and 0.944, respectively. The cutoff values were as follows: UDCA was 21.02 pmol/mL, CDCA was 56.77 pmol/mL, TCA was 187.49 pmol/mL, TUDCA was 22.64 pmol/mL, TCDCA was 643.25 pmol/mL, GUDCA was 72.71 pmol/mL, GCDCA was 1647.77 pmol/mL, and GCA was 619.79 pmol/mL.

ROC analysis was performed for 14 bile acids in the hepatitis and control groups. A total of 8 bile acids with AUC value >0.700 are shown in Figure 4.

#### 3.1.6. Correlations Between Bile Acids and Brunt Classification (Grading, Staging)

**Figure 5:**
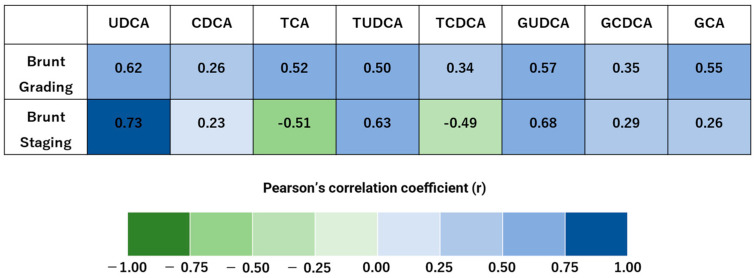
Bile acids show strong positive correlations with liver fibrosis indicators in MASLD patients. Pearson’s correlation coefficients for 8 bile acids are visualized as heat maps, illustrating significant relationships between Brunt Grading and Staging [32]. Brunt Grading consists of three items: steatosis, ballooning, and inflammation. Brunt Staging indicates the degree of fibrosis. Correlation strength is classified as follows: 0.7 < |r| < 1.0 indicates a strong correlation. Brunt Staging was strongly correlated with ursodeoxycholic acid (UDCA).

UDCA shows strong positive correlations with liver fibrosis indicators in MASLD patients (Figure 5).

## 4. Discussion

In this study, we profiled 14 plasma bile acids in the hepatitis and control groups using targeted LC-MS/MS metabolomics. Previous blood bile acid analyses of ALD, MASLD, and cirrhosis have not used employed LC-MS/MS [33,34,35].

The serum bile acid levels have been associated with cirrhosis [36,37,38]: higher GCA, GCDCA, TCA, and TCDCA in patients with hepatitis B virus cirrhosis [37,39]; and higher TCA, TCDCA, TUDCA, GCA, GCDCA, and GUDCA in alcoholic cirrhosis patients compared with controls [34]. Conjugated bile acids were thought to indicate liver dysfunction in cirrhosis and chronic hepatitis [37,40,41]. TCDCA and CDCA reportedly induce antiapoptotic proteins and mRNAs in hepatocytes [42]. It is believed that chronic cholestasis in the presence of GCDCA in mice induces profibrotic signals at the mRNA level, leading to excessive collagen deposition in liver tissue [43]. In this study, UDCA, CDCA, TCA, TUDCA, TCDCA, GUDCA, GCDCA, and GCA were significantly higher in the hepatitis group than in the control group. Consistent with the aforementioned reports, TCA, TUDCA, TCDCA, GUDCA, GCDCA, and GCA were elevated in the hepatitis group. The ROC analysis of 8 bile acids resulted in AUC values that appear to have high diagnostic capacity for hepatitis.

Continuous administration of UDCA may replace the more cytotoxic bile acids [44]. Administration of UDCA is unlikely to increase bile concentrations other than UDCA and TUDCA. In the intestine, UDCA binds to taurine to form TUDCA. We believe that patients in the hepatitis group received UDCA, which increased TUDCA concentrations and was consequently associated with liver damage.

Increased serum bile acid levels are considered more sensitive for detecting cirrhosis than liver function tests [45,46]. However, we have not found any reports examining the relationship of bile acids with hepatic reserve capacity. In this study, TUDCA and GCDCA showed a strong positive correlation in TBIL and DBIL. TCDCA and GCDCA were strongly and positively correlated with AST. Only GCDCA showed a strong correlation with hepatobiliary enzymes. GCDCA is suspected of being involved in cytotoxicity and our results suggest as much [47].

Serum bile acids are correlated with the CP score in cirrhotic patients [33,34] and with the MELD score in patients with alcoholic cirrhosis [34]. In this study, the CP score, FIB-4 index, and NFS showed a strong positive correlation in GCA. APRI showed a strong positive correlation with TCA, TCDCA, and GCA. MELD score showed a strong positive correlation with GCDCA. The present results indicate that these metabolites could be potential biomarkers for the transition from normal to cirrhotic conditions. Among them, GCDCA is strongly correlated with hepatic biliary enzymes and the MELD score. TBIL of the MELD score is elevated in patients with biliary cholestasis. And GCDCA, the major toxic component of bile acids, is elevated in patients with biliary cholestasis. Thus, the MELD score and GCDCA are associated with disease progression. In MASH and AH, bile acid pools are thought to be abnormal due to an impaired enterohepatic circulatory system, making bile acid metabolomics useful. GCDCA has been reported as a marker of liver damage [34,37,39], based on the results of this study. GCDCA may be the best marker of liver damage for early intervention. When GCDCA is elevated, early nutritional management with dietary guidance for MASH patients and abstinence from alcohol for AH patients can help control disease progression.

## 5. Conclusions

It is possible to analyze bile acids in plasma by LC-MS/MS. As UDCA, CDCA, TCA, TUDCA, TCDCA, GUDCA, GCDCA, and GCA were elevated, suggesting that TUDCA, TCDCA, and GCDCA are associated with liver function and may be used as predictive markers of cirrhosis. GCA, TCA, TCDCA, and GCDCA were associated with the degree of liver damage and could be used as predictive markers of cirrhosis. Among bile acids, GCDCA may be the best marker of liver damage for early intervention. Additional case studies are needed to validate these findings.

## Figures and Tables

**Table 1:** Clinical comparisons between the hepatitis and control groups.

Variable	Hepatitis, MASH, N = 8 Median (Min, Max)	Hepatitis, AH, N = 3 Median (Min, Max)	Controls, N = 8 Median (Min, Max)	MASH vs. AH	AH vs. Controls	MASH vs. Controls
Sex (Man/Female)	3/5	2/1	4/4	0.186	0.028	0.165
Age (year)	63 (54, 68)	52 (47, 52)	77 (34, 88)	0.675	0.219	0.674
BMI (Kg/m^2^)	24.5 (22.9, 35.3)	24.7 (21.9, 25.6)	23.0 (18.5, 29.5)	0.676	0.102	0.172
Diabetes mellitus	5	0	0	0.516	0.015	0.003
Ursodeoxycholic acid Administration	7	1	0	0.582	0.022	0.002
PLT (10^4^/μL)	4.8 (3.6, 9.1)	6.9 (5.4, 10.3)	25.9 (11.8, 41.9)	0.095	0.413	0.093
TBIL (mg/dL)	1.9 (0.5, 4.2)	4.6 (1.7, 11.3)	0.6 (0.4, 1.5)	0.095	0.014	0.006
DBIL (mg/dL)	0.3 (0.1, 0.8)	0.5 (0.3, 4.8)	0.06 (0.03, 0.2)	0.210	0.014	0.002
AST (U/L)	31 (21, 58)	24 (24, 64)	21 (13, 34)	0.464	0.051	0.015
ALT (U/L)	17 (7, 41)	16 (7, 17)	21 (10, 40)	0.346	0.182	0.527
γGTP (U/L)	26 (9, 101)	23 (20, 25)	29 (11, 157)	0.403	0.152	0.400
TCHOL (mg/dL)	117 (51, 184)	93 (65, 214)	192 (156, 239)	0.094	0.031	0.207
TG (mg/dL)	46 (22, 86)	26 (25, 28)	98 (59, 152)	0.531	0.413	0.172
Cholinesterase (U/L)	95 (23, 298)	76 (58, 268)	235 (217, 367)	0.144	0.065	0.462
PT-INR	1.4 (1.1, 1.7)	1.8 (1.2, 1.9)	1.0 (1.0, 1.1)	0.403	0.014	<0.001
ALB (g/dL)	2.9 (2.4, 3.6)	2.5 (2.2, 3.6)	4.0 (3.5, 4.5)	0.116	0.214	0.140
Creatinine (mg/dL)	0.9 (0.5, 1.5)	0.7 (0.6, 1.2)	0.8 (0.7, 2.9)	0.531	0.413	0.400
CP score	9 (8, 12)	11 (10, 12)	5 (5, 6)	0.244	0.007	<0.001
FIB-4 index	10.0 (3.7, 23.4)	8.0 (2.0, 11.7)	1.3 (0.2, 5.5)	0.338	0.066	0.001
NFS	3.0 (2.1, 6.1)	3.6 (0.08, 3.8)	−1.2 (−5.1, 1.0)	0.676	0.024	<0.001
APRI	17.3 (6.9, 33.0)	11.1 (5.8, 23.2)	2.0 (0.8, 7.2)	0.676	0.024	0.001
ALBI grade	−1.4 (−1.9, −1.3)	−0.9 (−1.8, −0.6)	−2.6 (−3.2, 2.3)	0.531	0.014	<0.001
MELD score	14.3 (8.6, 15.6)	14.9 (14.6, 23.9)	6.8 (3.4, 13.6)	0.210	0.014	0.002

**Table 2:** Plasma bile acid comparisons between the hepatitis and control groups.

Bile acids	Hepatitis, N = 11 pmol/mL Median (Min, Max)	Controls, N = 8 pmol/mL Median (Min, Max)	*p* Value
Ursodeoxycholic acid (UDCA)	389 (<40, 1102)	<40 (<40, <40)	0.003
Chenodeoxycholic acid (CDCA)	97 (<40, 1065)	<40 (<40, 376)	0.015
Taurocholic acid (TCA)	652 (<40, 4188)	<40 (<40, 168)	<0.001
Tauroursodeoxycholic acid (TUDCA)	1073 (<40, 4567)	<40 (<40, <40)	0.001
Taurochenodeoxycholic acid (TCDCA)	1522 (<40, 16867)	<40 (<40, 323)	0.001
Glycoursodeoxycholic acid (GUDCA)	6231 (<40, 20891)	20.9 (<40, 77)	0.007
Glycochenodeoxycholic acid (GCDCA)	7367 (157, 34807)	224.7 (48, 1046)	0.002
Glycodeoxycholic acid (GDCA)	<40 (<40, 294)	50.9 (<40, 126)	0.161
Glycocholic acid (GCA)	1231 (111, 2121)	<40 (<40, 366)	<0.001
Deoxycholic acid (DCA)	<40 (<40, 75)	<40 (<40, 104)	0.363
Cholic acid	<40 (<40, 116)	<40 (<40, 49)	0.877
Taurodeoxycholic acid	<40 (<40, 75)	<40 (<40, <40)	0.215
Lithocholic acid	<40 (<40, 110)	<40 (<40, <40)	0.394
7-Ketolithocholic acid	<40 (<40, <40)	<40 (<40, <40)	-

## Data Availability

All data are available from the corresponding author.

## Supplementary Materials

The following supporting information can be downloaded at: https://www.mdpi.com/article/10.3390/biomedicines13010078/s1, Table S1: Summary of MRM parameters and retention times for the targeted compounds.
