# Supplementary material for: Targeted Plasma Bile Acid Metabolomic Analysis in Metabolic Dysfunction-Associated Steatohepatitis and Alcoholic Hepatitis
Source: Biomedicines. 2024 Dec 31;13(1):78. doi: 10.3390/biomedicines13010078 (PMC11762544; doi:10.3390/biomedicines13010078)
Supplement: Supplementary file 1 [file biomedicines-13-00078-s001.zip › biomedicines-3383506-supplementary.pdf]

Supplementary Table S1. Summary of MRM parameters and retention times for the targeted compounds.

**Analyte**	**Precursor**	**Product**	**Collison (V)**	**Retention time (min)**
7-Ketolithocholic acid [M+H]+	373.3	355.3	−15	5.6
UDCA [M+H]+	357.3	161.1	−25	5.1
CDCA [M+H]+	357.3	161.1	−25	7.0
LCA [M+H]+	359.3	135.1	-25	9.0
TCA [M−H]−	514.3	514.3	25	4.2
TUDCA [M−H]−	498.3	498.2	28	3.7
TCDCA [M−H]−	498.3	498.2	28	5.6
TDCA [M−H]−	498.3	498.2	28	6.0
GUDCA [M−H]−	448.3	448.3	19	3.9
GCDCA [M−H]−	448.3	448.3	19	5.6
GDCA [M−H]−	448.3	448.3	19	5.9
GCA [M−H]−	464.3	464.3	17	4.3
CA [M−H]−	407.3	407.3	21	5.4
DCA [M−H]−	391.3	391.3	20	7.2
